# Supplementary material for: MEDALT: single-cell copy number lineage tracing enabling gene discovery
Source: Genome Biol. 2021 Feb 23;22:70. doi: 10.1186/s13059-021-02291-5 (PMC7901082; doi:10.1186/s13059-021-02291-5)
Supplement: Supplementary file 1 — Additional file 1: Fig. S1. Methodology of the framework. a. Illustration of minimal event distance (MED) calculation. b. Average lineage partitioning accuracy (LPA) on 100 simulation datasets without noise. c. Estimating lineage specific cumulative fold level (CFL). d. Estimating significance of CFL in an individual sample. e. AUC of non-random fitness-associated alterations (FAAs) detection based on LSA, permutated SCCN matrix rather than reconstructing tree, GISTIC test and one-side Wilcoxon signed-rank test on 100 simulation datasets without noise. f. Identification of non-random fitness-associated CNAs in a cohort of samples. g. Identification of parallel evolution CNAs in an individual sample. Fig. S2. The efficiency of MEDALT based on 9 × 3 × 20 simulation datasets with the population size from 400 to 2000, genome size from 100 to 1000. Fig. S3. Simulation and evaluation of CNA evolution model. a. Illustration of simulated genomic structural rearrangements in the evolution of a tumor. K represents the number of CNAs during ∆t period. r represents the number of adjacent regions which are affected by a CNA. TD: tandem duplication. TER: terminal deletion. DEL: interstitial deletion. BFB: breakage fusion bridge. b. Simulated and inferred copy number evolution distance between two genomes. Compared with MED are commonly used distance metrics Hamming, Euclidean and Manhattan. c. The AUC for identifying FAAs based on different combinations of models. Wilcox represents one-side Wilcoxon signed-rank test. d. The effects of noise on FAAs detection. Fig. S4. SCCN profile of TNBC patient KTN102. Each row represents a cell from pre-, mid-, or post-treatment. Fig. S5. Average distance between root node and cells from pre-, mid- or post-treatment based on MEDALT, maximal parsimony (MP), neighbor-joining (NJ) and maximum likelihood tree. FC refers to the fold changes between the average distance to root of the mid−/post- cells and that of the pre-treatment cells. Fig. S6. St [file 13059_2021_2291_MOESM1_ESM.docx]

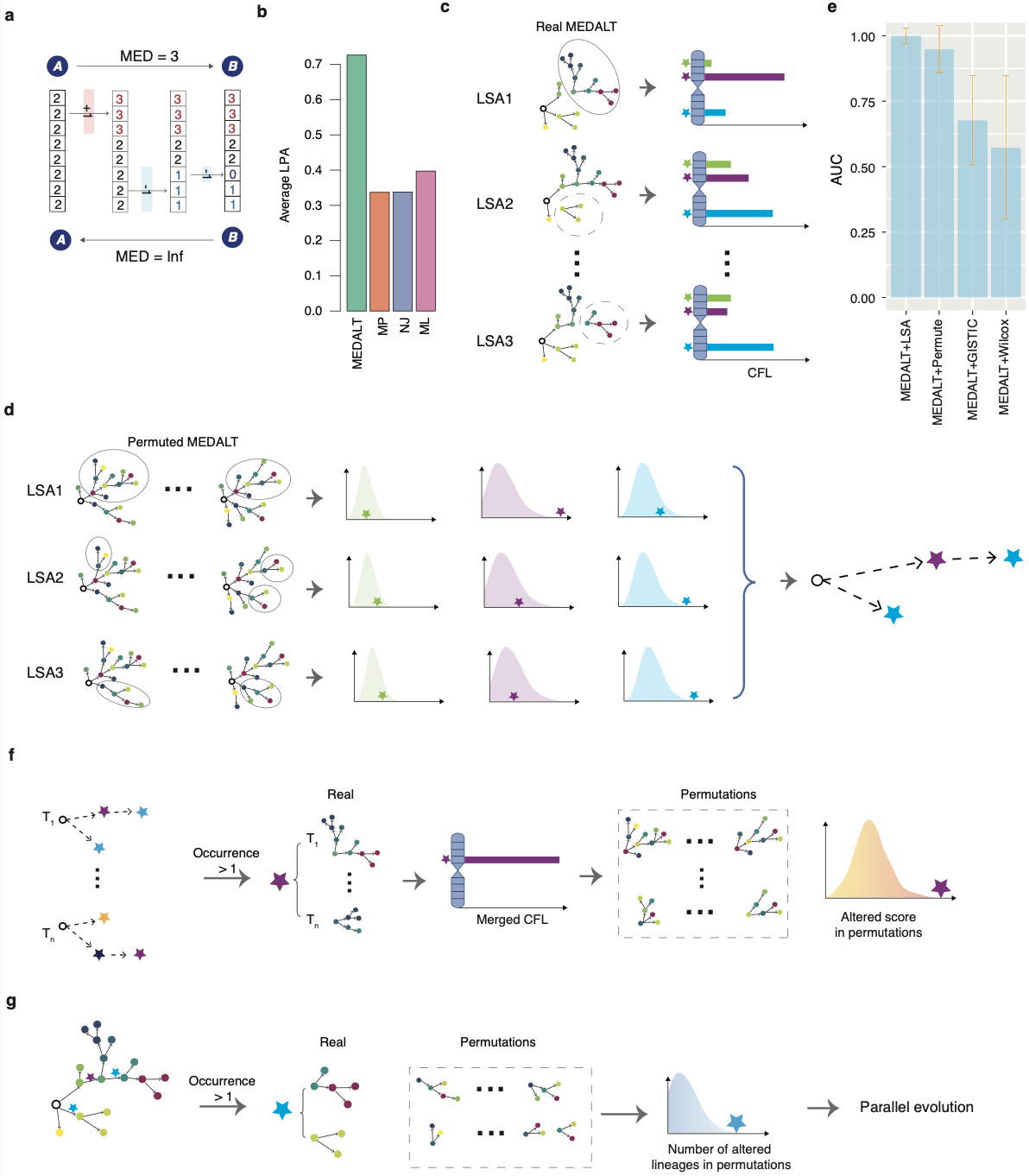


**Fig S1** Methodology of the framework. **a.** Illustration of minimal event distance (MED) calculation. **b.** Average lineage partitioning accuracy (LPA) on 100 simulation datasets without noise. **c**. Estimating lineage specific cumulative fold level (CFL). **d.** Estimating significance of CFL in an individual sample. **e.** AUC of non-random fitness-associated alterations (FAAs) detection based on LSA, permutated SCCN matrix rather than reconstructing tree, GISTIC test and one-side Wilcoxson signed-rank test on 100 simulation datasets without noise. **f.** Identification of non-random fitness-associated CNAs in a cohort of samples. **g.** Identification of parallel evolution CNAs in an individual sample.


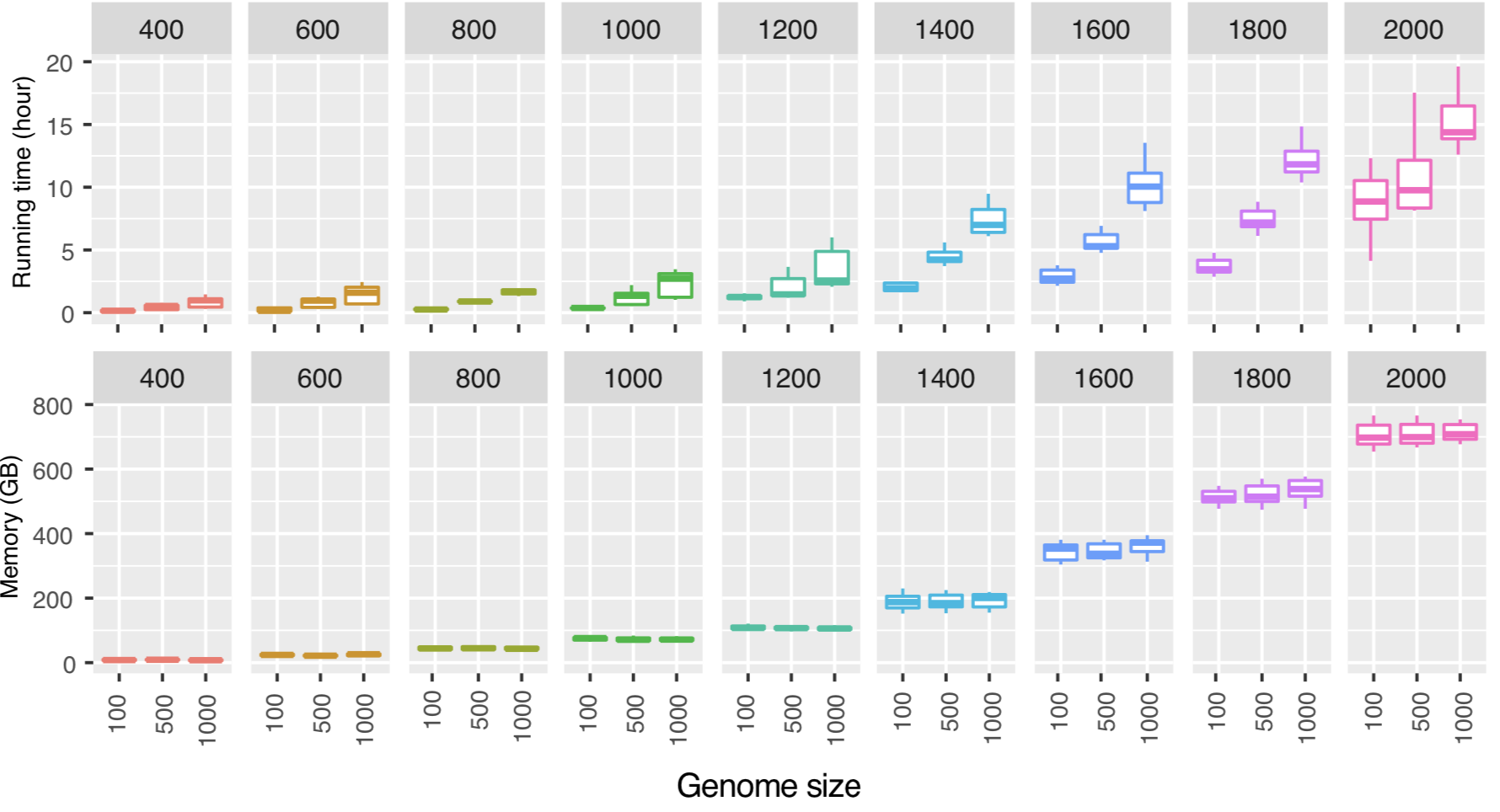


**Fig S2** The efficiency of MEDALT based on $9\times3\times20$ simulation datasets with the population size from 400 to 2000, genome size from 100 to 1000.

**
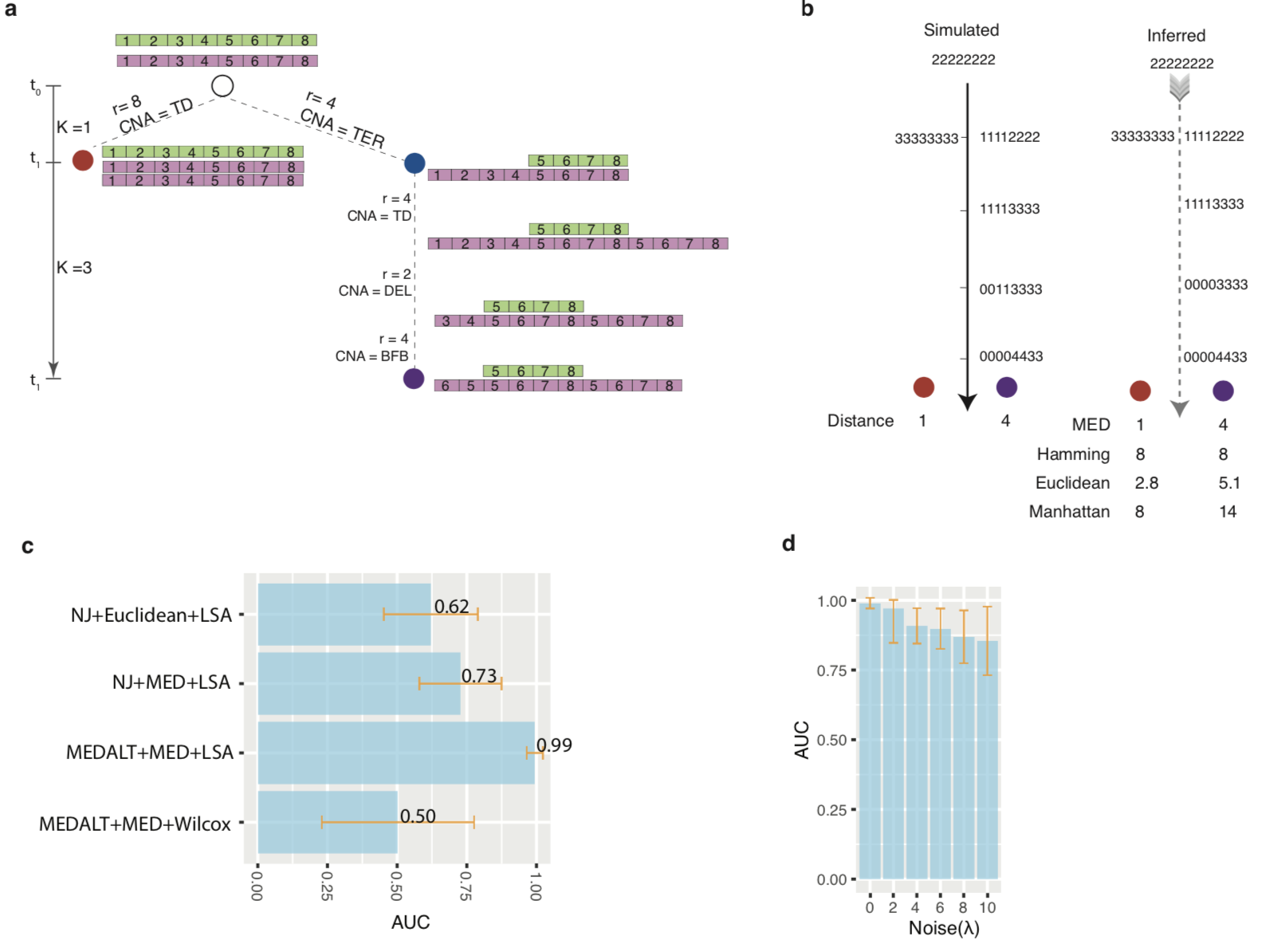
**

**Fig S3** Simulation and evaluation of CNA evolution model. **a.** Illustration of simulated genomic structural rearrangements in the evolution of a tumor. K represents the number of CNAs during $\Delta t$ period. r represents the number of adjacent regions which are affected by a CNA. TD: tandem duplication. TER: terminal deletion. DEL: interstitial deletion. BFB: breakage fusion bridge. **b**. Simulated and inferred copy number evolution distance between two genomes. Compared with MED are commonly used distance metrics Hamming, Euclidean and Manhattan. **c.** The AUC for identifying FAAs based on different combinations of models. Wilcox represents one-side Wilcoxon signed-rank test. **d.** The effects of noise on FAAs detection.


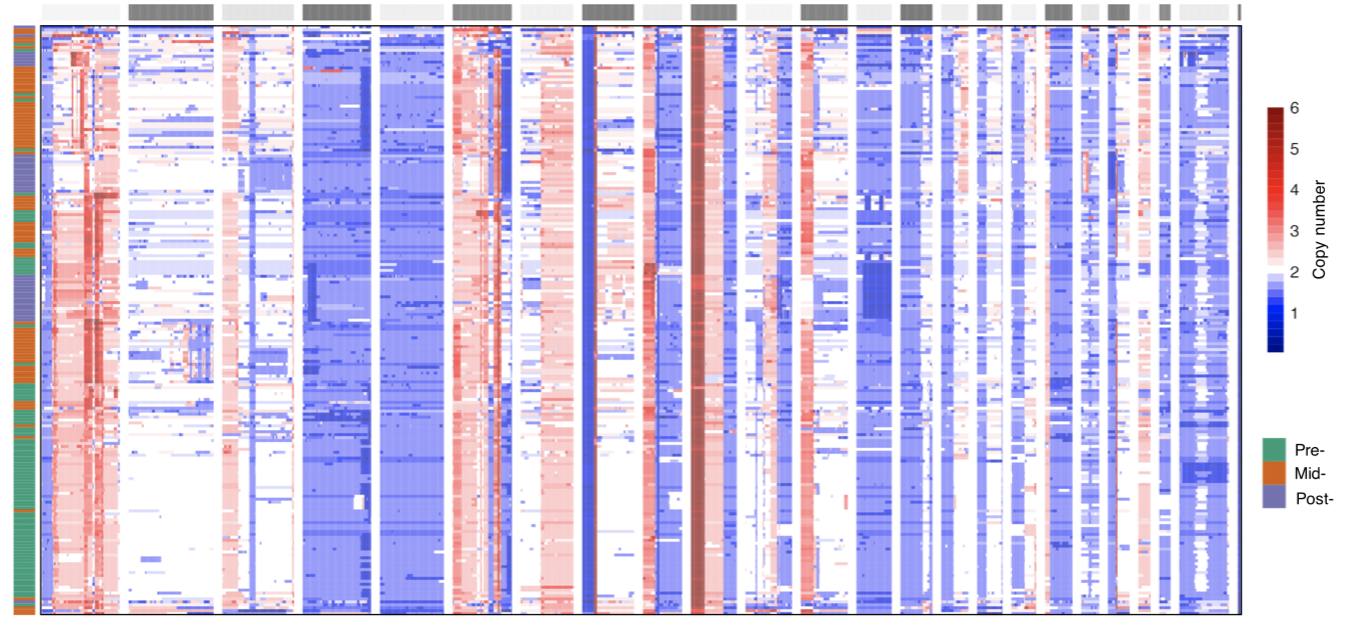


**Fig S4** SCCN profile of TNBC patient KTN102. Each row represents a cell from pre-, mid-, or post-treatment.

**
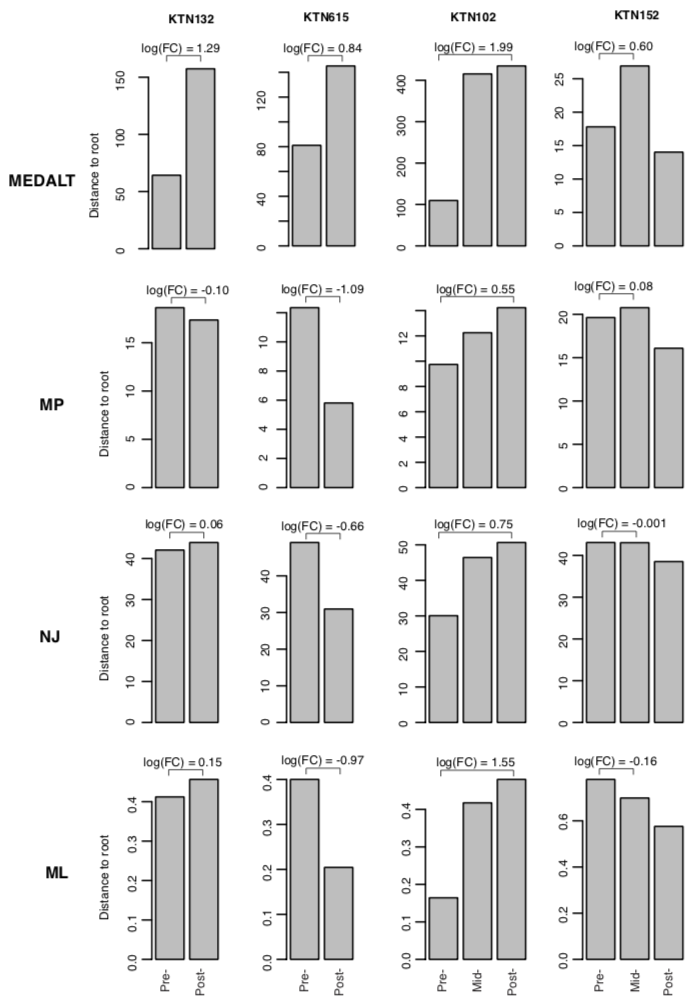
**

**Fig S5** Average distance between root node and cells from pre-, mid- or post-treatment based on MEDALT, maximal parsimony (MP), neighbor-joining (NJ) and maximum likelihood tree. FC refers to the fold changes between the average distance to root of the mid-/post- cells and that of the pre-treatment cells.


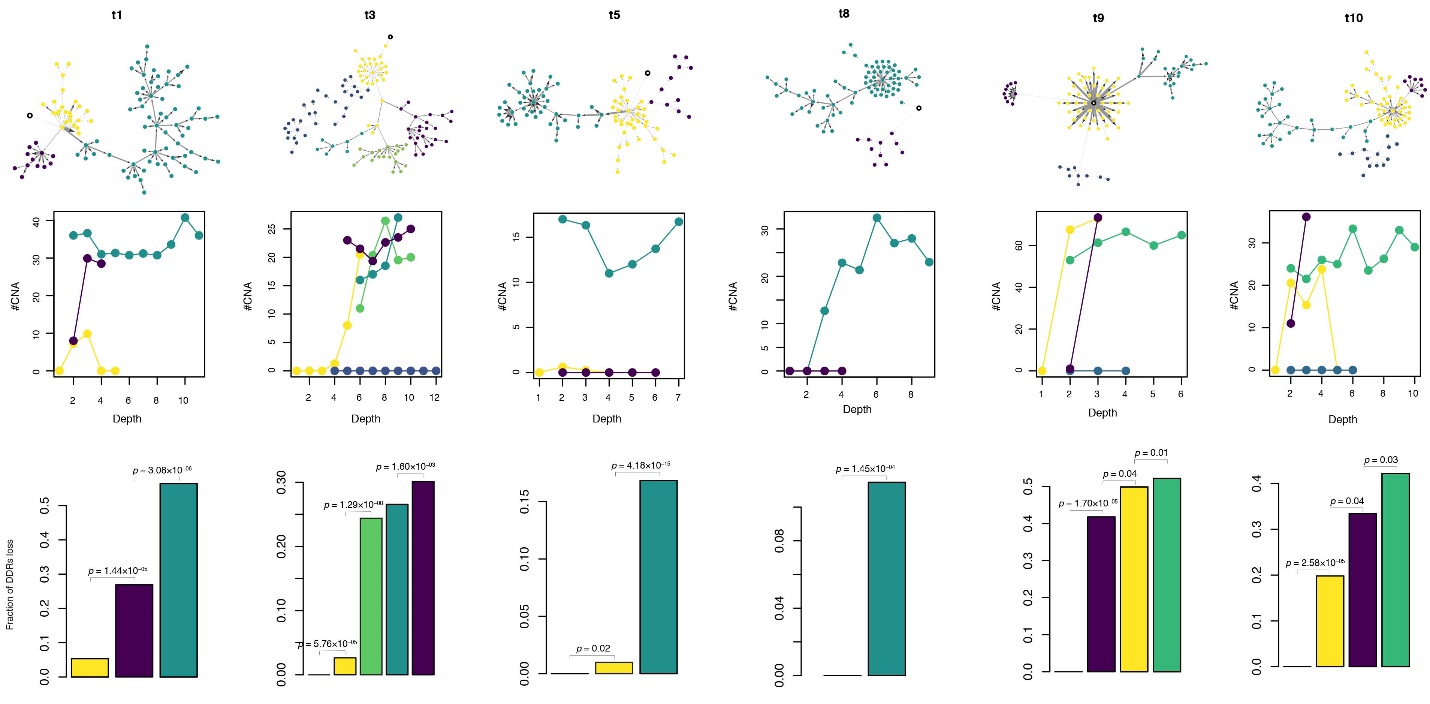


**Fig S6** Stratified average CNA rates and fractions of DDR genes loss among lineages (distinguished by colors) in 6 primary TNBC samples


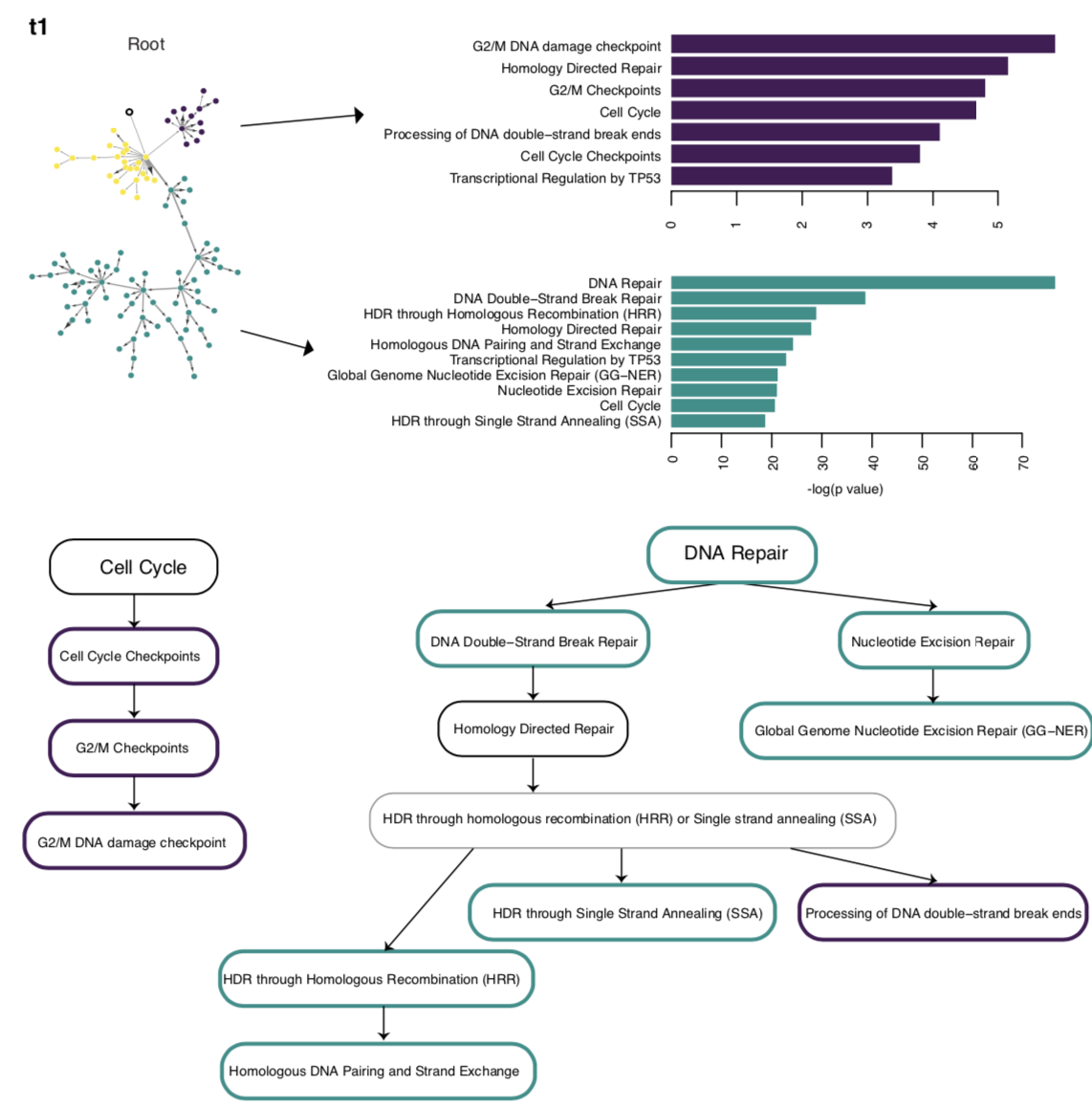
­

**Fig S7** Gene set enrichment analysis (GSEA) for genes identified by LSA in patient t1. Colors correspond to branches.


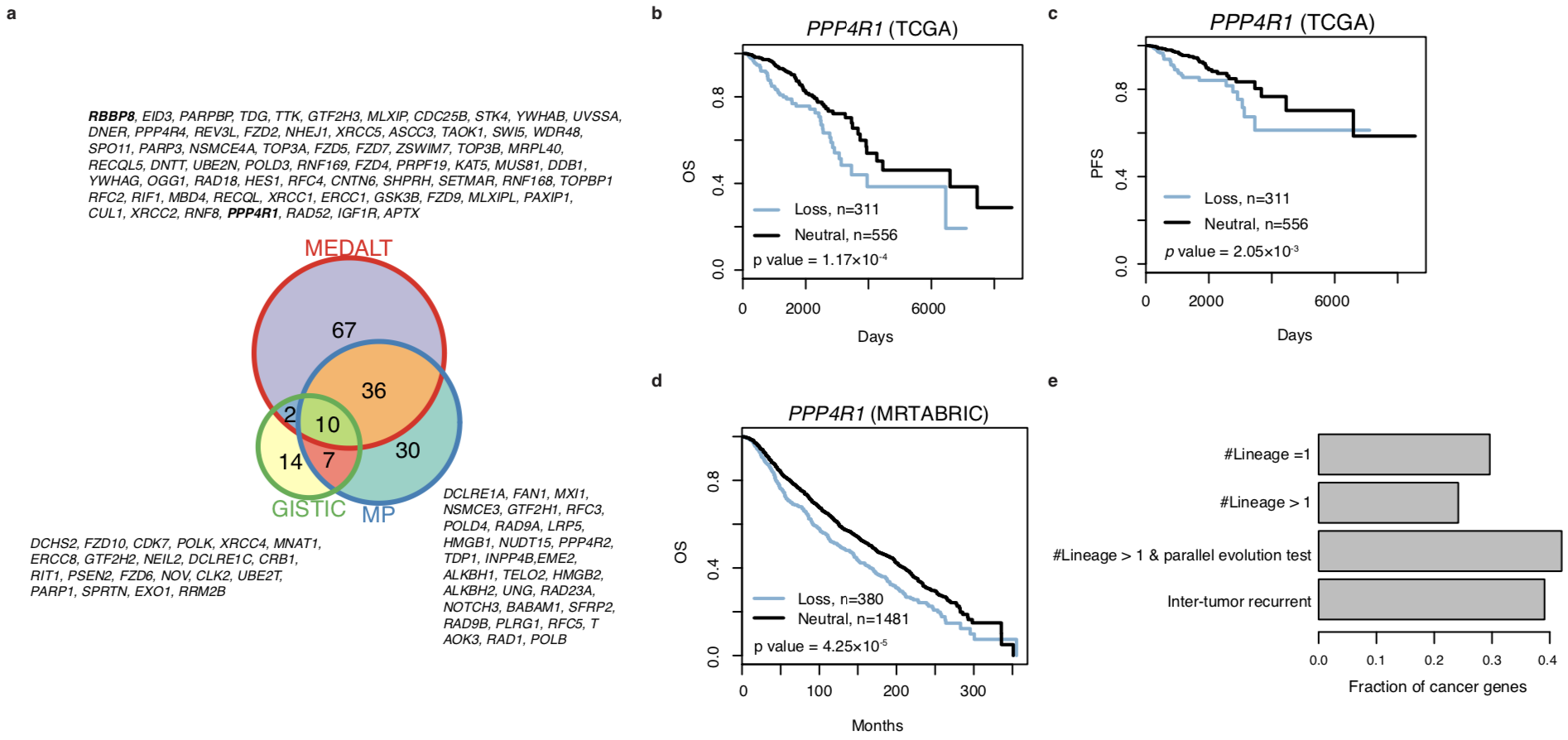


**Fig S8** Significant genes identified through cohort LSA from the TNBC scDNA-seq data. **a.** Venn diagram of the genes identified by the MEDALT, MP and GISTIC but not reported in oncoKB, COSMIC and intOGen. **b.** Overall survival (OS) analysis of breast cancer patients in TCGA. **c**. Progression free survival (PFS) analysis of breast cancer patients in TCGA. **d**. Overall survival analysis of breast cancer patients in the METABRIC. **e.**  The fraction of cancer genes overlapping with events which were significant in single lineage (#Lineage = 1), multiple lineages (#Lineage > 1), parallel evolution test ((#Lineage > 1& PLSA < 0.001) and cohort LSA (inter-tumor recurrent).


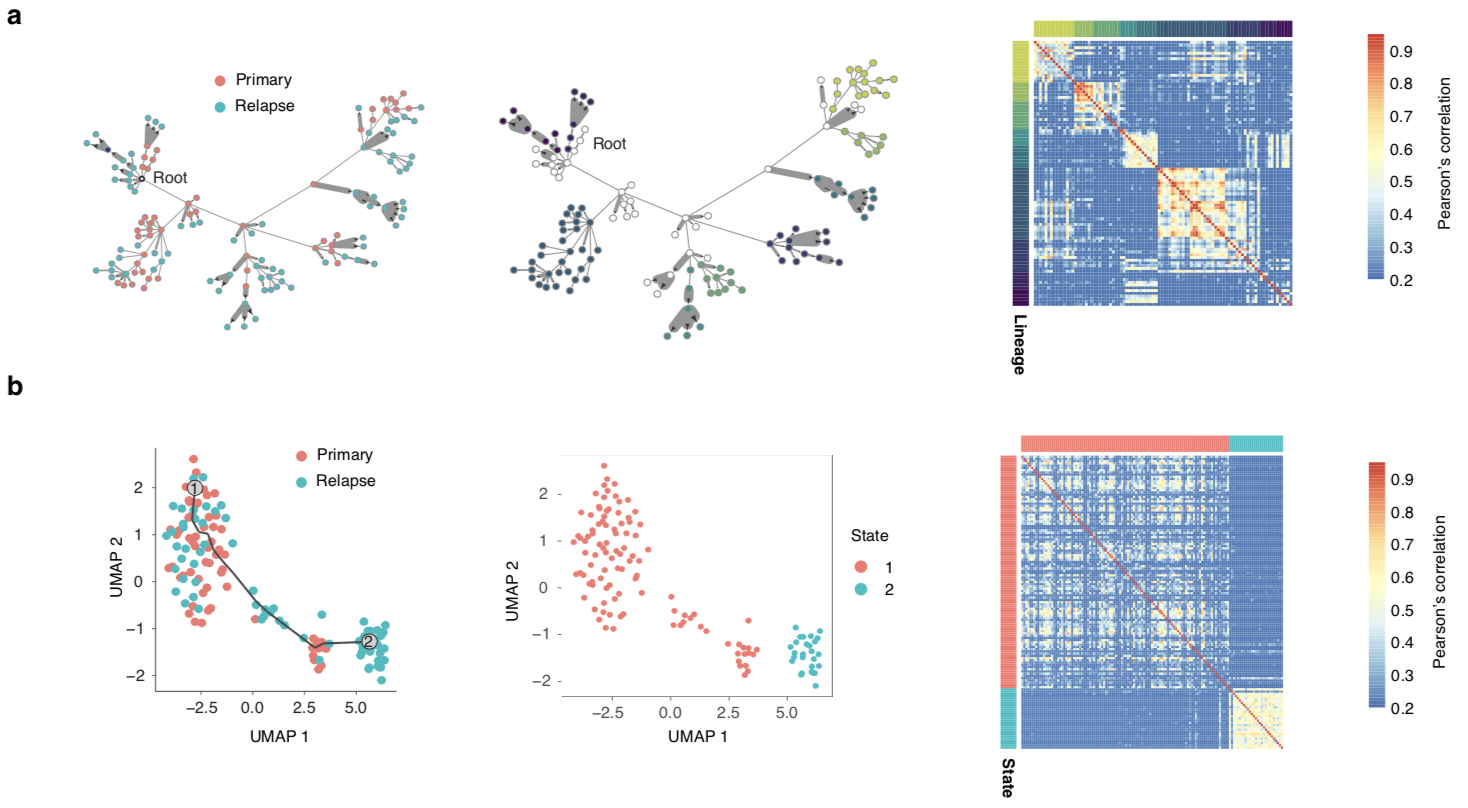


**Fig S9** Results of multiple myeloma patient 60359. **a.** Inferred MEDALT and heatmap based on Pearson’s correlation of the inferCNV profiles between cells ordered by lineages in MEDALT. **b.** Inferred trajectory from Monocle and heatmap of Pearson’s correlation of the inferCNV profiles between cells ordered by states defined by Monocle.

**Table S1 The algorithm for minimal event distance (MED) inference**

**Table S2 The algorithm for rooted directed minimal spanning tree reconstruction**

Where *m*(*u*) is minimum weight of all edge going into *u, me*(*u*) as the edge that has the minimum weight of all edges going into *u.*

**Table S3 Information on the TNBC data**

| **Patient ID** | **Sample size** | | |
| --- | --- | --- | --- |
|  | **Primary/Pre-treatment** | **Mid-treatment** | **Post-treatment** |
| t1 | 100 |  |  |
| t2 | 65 |  |  |
| t3 | 110 |  |  |
| t4 | 52 |  |  |
| t5 | 89 |  |  |
| t6 | 90 |  |  |
| t7 | 68 |  |  |
| t8 | 82 |  |  |
| t9 | 85 |  |  |
| t10 | 92 |  |  |
| t11 | 100 |  |  |
| t12 | 48 |  |  |
| KTN206 | 45 |  |  |
| KTN126 | 30 |  |  |
| KTN129 | 33 |  |  |
| KTN302 | 46 |  |  |
| KTN615 | 16 | 34 |  |
| KTN132 | 43 | 31 |  |
| KTN102 | 88 | 76 | 37 |
| KTN152 | 43 | 28 | 40 |

**Table S4 Annotation of the broad CNAs identified in TNBCs based on literature**

| **CNA** | **Region** | **Method** | **Cancer** | **Functional description** | **PMID** |
| --- | --- | --- | --- | --- | --- |
| Loss | Chr5q | MEDALT, MP | **Breast cancer**, prostate cancer, cervical cancer | Tumor growth and metastatic progression | 29562176, 29662167, 30022638 |
|  | Chr2q | MEDALT, MP | **Breast cancer** | More aggressive tumor type, moderately/poorly differentiated tumors. | 10861509, 8818658, 13680524 |
|  | Chr9q | MEDALT | **Breast cancer**, bladder cancer, head and neck cancer, lung cancer, liver cancer | Invasive tumor lesions, | 8550238, 11438741, 16149093, 8118798, 8306323, 10209942 |
|  | Chr3p | MP | **Breast cancer**, squamous cancer, ovarian cancer, Vulvar Carcinoma | reduces cell proliferation, poor survival | 29622463, 29558370, 30279231, 27170308, 10850424 |
|  | Chr17p | MEDALT | **Breast cancer** |  | [26328251](https://www.ncbi.nlm.nih.gov/pubmed/26328251), [25481507](https://www.ncbi.nlm.nih.gov/pubmed/25481507), 13680524 |
|  | Chr4p | MP | Meningiomas, metastatic thymic adenocarcinoma |  | 31591222, 28506304 |
|  | Chr21q | MEDALT, MP | **Breast cancer**, non-small cell lung cancer, oral cancer susceptibility | Associated with tumor suppressor | 13680524,12735585, 9743305, 9790505, 13680524, 9523199, 11259088 |
|  | Chr7p | MEDALT, MP | **Breast cancer** |  | 10861509 |
|  | Chr23p | MP | **Breast cancer** |  | 20101236 |
|  | Chr10q | MEDALT | **Breast cancer**, Brain tumor, head and neck cancer, oligodendrogliomas | Invasive phenotype, poor prognostic | 22429330, 14676808, 10404099, 11438486 |
|  | Chr16q | MEDALT, MP | **Breast cancer** | Invasive phenotype, tumor suppressor | 29558370, 19156836, 13680524, 14976537, 8971163, 16280054, 21706489, 23348384 |
|  | Chr22q | MEDALT, MP | Breast cancer, colorectal cancer, glioblasma, gastrointestinal stromal tumor, insulinoma | Tumor suppressor | 10850424, 13680524, 29665859, 14712485, 10861509, 15580284, 11739439 |
|  | Chr8p | MEDALT, MP | **Breast cancer**, Prostate cancer | Poor prognosis | 27611943, 13680524 |
|  | Chr15q | MEDALT, MP | **Breast cancer**, bladder cancer | More aggressive tumor type, metastatic carcinoma | 8649814, 14633686 |
|  | Chr4q | MEDALT | **Breast cancer**, colon cancer, multiple myeloma | Worse outcome | 9743305, 21717218, 10446964, 17077331 |
|  | Chr12q | MEDALT | **Breast cancer**, pancreatic cancer |  | 19602461, 15300227 |
|  | Chr20p | MEDALT | Colorectal cancer |  | 12490103, 19359472 |
|  | Chr6q | MEDALT, MP | **Breast cancer** | Tumor suppressor | 9155053, 10778973 |
|  | Chr17q | MEDALT | **Breast cancer,** non-small cell lung cancer |  | 7671234 |
|  | Chr5p | MEDALT | **Breast cancer** |  | 16570289 |
|  | Chr18q | MEDALT, MP | **Breast cancer** |  | 13680524 |
| Gain | Chr8q | MEDALT, GISTIC, MP | **Breast cancer**, melanoma, kidney cancer | More aggressive tumor phenotype, high risk of distant metastases and greater tumor size. | 10861509, 11514955, 22605478, 31645765 |
|  | Chr6p | MEDALT, MP | **Breast cancer**, melanoma, liver cancer, ovarian cancer, glioblastoma | More aggressive tumor phenotype,  advanced or metastatic disease,  poor prognosis. | 10861509, 11514955, 16790693 |
|  | Chr3q | MEDALT, MP | **Breast cancer**, lung cancer | More aggressive tumor phenotype. | 10861509, 18317062 |
|  | Chr7q | MEDALT, MP | **Breast cancer**, colorectal cancer | metastasis | 29558370, 26894854 |
|  | Chr10p | MEDALT, MP | colon cancer |  | 29580161 |
|  | Chr20q | MP | Colorectal cancer, pancreatic cancer |  | 22860045, 29169336, 6568296, 28991255, 29991641, 26894854 |
|  | Chr9p | MEDALT, MP | Multiple cancer |  | 29212506 |
|  | Chr12p | MEDALT, MP | **Breast cancer**, pancreatic cancer | Docetaxel resistance, Carboplatin Sensitivity, aggressive phenotype | 31213465, 30873387 |
|  | Chr5p | MP | Cervical cancer, head and neck cancer, renal cell carcinoma |  | 18559093, 31427592, 19521957 |
|  | Chr6q | MEDALT | **Breast cancer** |  | 17925008, 24969692 |
|  | Chr16p | MP |  |  |  |
|  | Chr1q | GISTIC | **Breast cancer** |  | 14976537, 17060936 |
|  | Chr10q | MP |  |  |  |
|  | Chr12q | MP | Classical lobular carcinomas |  | 18473330 |
|  | Chr14q | MEDALT, MP |  |  |  |
|  | Chr18p | MEDALT, MP |  |  |  |
|  | Chr2q | MP |  |  |  |
|  | Chr20p | MP | **Breast cancer** |  | 12755492 |
|  | Chr4q | MP |  |  |  |
|  | Chr11q | MEDALT, MP |  |  |  |
|  | Chr16q | MEDALT |  |  |  |

**Table S5 Information on the scRNA-seq data**

| **Cancer** | **Patient ID** | **Sample size** | |
| --- | --- | --- | --- |
|  |  | **Primary** | **Metastasis/Relapse** |
| HNSCC | HN5 | 112 | 20 |
|  | HN6 | 80 | 44 |
|  | HN20 | 572 | 90 |
|  | HN25 | 61 | 148 |
|  | HN26 | 127 | 140 |
|  | HN28 | 70 | 68 |
| OV | HG1 | 227 | 250 |
|  | HG2F | 253 | 165 |
|  | HG3 | 25 | 6 |
|  | LG2 | 34 | 24 |
| OSCC | HN120 | 278 | 270 |
|  | HN137 | 413 | 155 |
| MM | 60359 | 51 | 82 |
|  | 27522 | 915 | 85 |
|  | 47491 | 640 | 596 |
|  | 56203 | 931 | 69 |
|  | 57075 | 177 | 823 |
|  | 58408 | 112 | 225 |
|  | 59114 | 33 | 323 |
|  | 81012 | 421 | 512 |
