# Supplementary material for: MEDALT: single-cell copy number lineage tracing enabling gene discovery
Source: Genome Biol. 2021 Feb 23;22:70. doi: 10.1186/s13059-021-02291-5 (PMC7901082; doi:10.1186/s13059-021-02291-5)
Supplement: Supplementary file 6 — Additional file 6. [file 13059_2021_2291_MOESM6_ESM.pdf]

Appendix

Proof of correctness

Here we provide a theoretical proof and running time analysis for the algorithm proposed in Table S1

We define the problem as follows, $X = [x_1, \ldots, x_m], Y = [y_1, \ldots, y_m]$ are two integer arrays of length m. The entries x_i and y_i indicate the number of copies of position i in cell X and Y. For simplicity, we present the proof for a single chromosome, the results can be easily extended to the case of multiple chromosomes.

Then we define an operation, or event, acting on array X increases (amplification) or decreases (deletion) value by 1 in a contiguous segment of X. Then Minimal Evolution Distance (MED) between X and Y is the minimal number of events that transforms X into Y. We added an additional restriction that once a position l has been lost, i.e. $x_l = 0$, it can never be regained or deleted. Thus, MED equals to infinity if the copy number at any site is going from 0 to any other number.

For any series of event that transforms X into Y, we can describe that sequence by $C = (c_1, \ldots, c_t)$, in which $c_i = (s_i, h_i, w_i)$, representing an event $w_i \in \{1, -1\}$ is affecting position from s_i to h_i.

Then we prove the correctness of the algorithm with the following definitions and propositions.

Definition 1 A sequence C is well-ordered if its starting position are non-decreasing and there are no opposite events affecting the same position. i.e.

$$\forall i < j, s_i \leq s_j$$

$$\nexists c_i, c_j \in C, k \in [1, m] \text{ s.t. } w_i = 1, w_j = -1, k \in [s_i, h_i], k \in [s_j, h_j]$$

Proposition 1 There exists an optimal solution that is well-ordered.

Proof: We prove by showing that any optimal solution can be transformed to a well-ordered sequence with the same length.

First we start by making the observation that C is commutative. Since C is a legal sequence of action, any permutation of it will still transform X to Y, as the same set of actions are performed at each position. This directly comes from the commutative quality of addition. This allows us to sort C to make its starting point non-decreasing.

Then we show that we can remove opposite actions on same position without changing the length of the sequence C. Assume we have two opposite action c_i, c_j that are overlapping. Without loss of generality, we assume $s_i \leq s_j$, then

Case 1 $s_i \leq s_j \leq h_i \leq h_j$. Then the action $(s_i, h_i, w_i), (s_j, h_j, w_j)$ is equivalent to $(s_i, s_j, w_i), (h_i, h_j, w_j)$ which is no longer overlapping.

Case 2 $s_i \leq s_j \leq h_j \leq h_i$. Then the action $(s_i, h_i, w_i), (s_j, h_j, w_j)$ is equivalent to $(s_i, s_j, w_i), (h_j, h_i, w_i)$ which is no longer overlapping.

By repeating the above transformation on every overlapping pair of actions in C, we can get an equivalent C' with the same length.

We've proved that we can transform an optimal solution C to C' of the same length which is well-ordered. So there exists an optimal solution that is well-ordered.

Proposition 2 For any well-ordered solution, $\sum_{i=1}^{t} h_i - s_i + 1 = |X - Y|$.

Proof: Since there is no opposite action on any given position, the total number of change happened at position i is $|x_i - y_i|$, so the total amount of change is $|X - Y|$. And for each action, position from s_i to h_i have been changed by 1, which is $h_i - s_i + 1$, in total per action. Since C transforms X into Y, we have $\sum_{i=1}^{t} h_i - s_i + 1 = |X - Y|$.

Definition 2 Let X^i represent X after first i actions in C, x_j^i represent the jth element in X^i.

Proposition 3 For any well-ordered solution C, s_i is the smallest different element between X^{i-1} and Y. i.e. $s_i = k$, which is the smallest element such that $x_k^{i-1} \neq y_k$

Proof: Since C is well-ordered, we have that $s_i \leq s_j \forall i < j$. If we assume s_i is not the smallest element between X^{i-1} and Y, then exists $k < s_i$, $x_k^{i-1} \neq y_k$. Because $X^t = Y, x_k^t = y_k$, then there is an action c_j after c_i that make $x_k^j = y_k$, since it includes the kth position, $s_j \leq k$. Then $s_j \leq k < s_i, i < j$, contradiction. So s_i is the smallest different element.

Definition 3 Let δ_i be the difference between X^i and Y i.e. $\delta_i = |X^i - Y|$

Proposition 4 For optimal well-ordered solution C^* and the solution given by our algorithm C. we have $\delta_i \leq \delta_i^*$ for all i. (here δ_i^* represents the difference after the first i steps in C^*)

Proof: Let i be the smallest number such that $c_i \neq c_i^*$, meaning the first different action between our solution and the optimal solution. Then we have $\delta_{i-1} = \delta_{i-1}^*, s_i = s_i^*$. (since by our algorithm, s_i is the first different position, which is same position given by C^* according to Prop 2). Then since our algorithm chooses the longest possible sub-sequence to change, our action will be at least as long as c_i^*. So after the ith step, we have $\delta_i \leq \delta_i^*, s_{i+1} \geq s_{i+1}^*$.

Then we show for every following step, we can maintain $\delta_j \leq \delta_j^*, s_{j+1} \geq s_{j+1}^*$. This is because assuming $c_j^* = (s_j^*, h_j^*, w_j^*)$, we can do at least as good as $c_j = (s_j, h_j^*, w_j^*)$ if that is the longest subsequence our algorithm finds, which results in $\delta_j = \delta_j^*, s_{j+1} = s_{j+1}^*$, still satisfying the less equal constraint.

Proposition 5 Our algorithm is optimal by the "greedy stays ahead" proving scheme.

Proof: Since we showed $\delta_i \leq \delta_i^*$ for all i, and we know the last step gives us $\delta_t \leq \delta_t^* = 0$. We know our solution is at as short as the optimal solution. Since the optimal solution is already the shortest solution, we proved our solution is also optimal.

Runtime Analysis

The algorithm (Table S1) solves optimal CNT in time $O(m)$, where m is the length of input array.

Input two arrays $X = [x_1, x_2, \ldots, x_m]$ and $Y = [y_1, y_2, \ldots, y_m]$, we assume the CNT is from X to Y.

We set initial $MED = 0$.

If $\exists i (1 \leq i \leq m)$ such that $x_i = 0$ and $y_i \neq 0$, $MED = \infty$. This is performed in linear time.

Otherwise, we calculate the distance $D = Y - X = [d_1, d_2, \ldots, d_m]$. This is performed in constant time $O(m)$.

For each position i, $|d_i|$ events are needed from X to Y. If $d = 0$, we skip the corresponding positions. We find a sequence of events $C = (c_1, c_2, \ldots, c_t), c_j = (s_j, h_j, w_j)$ such that $d_(s_j), \ldots, d_(h_j)$ corresponding to the longest contiguous entries having the same change direction. $w_j = \max(|d_{s_j}|, \ldots, |d_{h_j}|)$. Thus, $MED = \sum_{j=1}^{t} w_j$. The MED is calculated in linear time $O(m)$.

Therefore, the algorithm runs in linear time $O(m)$.
