# Supplementary material for: MEDALT: single-cell copy number lineage tracing enabling gene discovery
Source: Genome Biol. 2021 Feb 23;22:70. doi: 10.1186/s13059-021-02291-5 (PMC7901082; doi:10.1186/s13059-021-02291-5)
Supplement: Supplementary file 7 — Additional file 7. Review history. [file 13059_2021_2291_MOESM7_ESM.docx]

This manuscript was previously reviewed at another journal, and we are unable to share those reviews. We secured one of the original reviewers to assess the revisions.

**Reviewer 1**

I have reviewed this paper before and I am Reviewer #1 in the point- to-point response.

I will only comment on the points I raised in the previous review and how they have been addressed by the authors.

1. Novelty and comparison to existing approaches.

I find the authors' response somewhat evasive. While all relevant papers are now cited on p4 of the revised manuscript, the authors still do not really engage with the previous literature even though their own approach is very similar. For example, Zeira et al (the closest competitor and ref 34 in the paper) just gets mentioned on p4 as having the same asymptotic bound without stating that the only difference is the way 0s are being handled.

This is a strong paper and a lot of work is being presented and it wouldn't hurt to be more direct in addressing the fact that the idea of a minimum event distance is not new. This can be easily done with two changes:

(i) in the last paragraph of the introduction at "A few new .." start a new paragraph with something like "To overcome these challenges, previous studies introduced the minimum event distance. Our approach extends this research direction" and describe refs 32, 33, 34. [Also see point 6 below]

(ii) in the results: Asymptotic bounds are one thing, but how does the actual performance differ between MEDALT and the Zeira method? Could the authors show that while their idea is pretty much the same as Zeira's and leads to the same results, at least their algorithm and its implementation are faster?

2. How much do the different part so the algorithm contribute

This is a robust response.

3. Stability to noise

Also a robust response

4. Impact of confounders

Out-of-scope is a fair response

5. Whole genome duplications

Also fair response

6. Potential biases in LSA

I agree with the authors that LSA is the key contribution of this paper. Coming back to point 1, shouldn't this contribution be worked out better in the intro? For example, after the paragraph I propose on minimum event distances, you could say something like "Having this new distance was a good step forward, but it is still a big problem how to identify significant patterns/genes in the trees. This is where we our new method comes in". That would re-focus the intro on the gene discovery that is in the title and not on the tree reconstruction (as it is now). However, you will then have to survey this field too and explain what you mean by the sentence "it is safe to say that given the same amount of computation, we are exploring more biologically meaningful space that current approaches."

7. Code

The code base has indeed improved. However, gencode_v38_gene_pos.txt seems corrupt and only contains chromosome 1.

Very minor additional point in Figure 1c: permutions -> permutations

Florian Markowetz

**Authors’ response**

1.	Novelty and comparison to existing approaches.

I find the authors' response somewhat evasive. While all relevant papers are now cited on p4 of the revised manuscript, the authors still do not really engage with the previous literature even though their own approach is very similar. For example, Zeira et al (the closest competitor and ref 34 in the paper) just gets mentioned on p4 as having the same asymptotic bound without stating that the only difference is the way 0s are being handled. 

This is a strong paper and a lot of work is being presented and it wouldn't hurt to be more direct in addressing the fact that the idea of a minimum event distance is not new. This can be easily done with two changes:
(i) in the last paragraph of the introduction at "A few new .." start a new paragraph with something like "To overcome these challenges, previous studies introduced the minimum event distance. Our approach extends this research direction" and describe refs 32, 33, 34. [Also see point 6 below]

Addressed as suggested.

(ii) in the results: Asymptotic bounds are one thing, but how does the actual performance differ between MEDALT and the Zeira method? Could the authors show that while their idea is pretty much the same as Zeira's and leads to the same results, at least their algorithm and its implementation are faster?   

Thanks for the kind suggestion. In the revised manuscript, we acknowledged that the MEDALT part of our approach is similar to Zeira et al. in principle. However, Zeira et al. did not release any software implementation that would allow us to make the requested comparison.

6.	Potential biases in LSA

I agree with the authors that LSA is the key contribution of this paper. Coming back to point 1, shouldn't this contribution be worked out better in the intro? For example, after the paragraph I propose on minimum event distances, you could say something like "Having this new distance was a good step forward, but it is still a big problem how to identify significant patterns/genes in the trees. This is where we our new method comes in". That would re-focus the intro on the gene discovery that is in the title and not on the tree reconstruction (as it is now). However, you will then have to survey this field too and explain what you mean by the sentence "it is safe to say that given the same amount of computation, we are exploring more biologically meaningful space that current approaches."

Great suggestions. We have updated the paragraph in the Introduction as suggested.

7.	Code

The code base has indeed improved. However, gencode_v38_gene_pos.txt seems corrupt and only contains chromosome 1.

Very minor additional point in Figure 1c: permutions -> permutations

Fixed
